# The Role of Chromosome X in Intraocular Pressure Variation and Sex-Specific Effects

**DOI:** 10.1167/iovs.61.11.20

**Published:** 2020-09-14

**Authors:** Mark J. Simcoe, Anthony P. Khawaja, Omar A. Mahroo, Christopher J. Hammond, Pirro G. Hysi, for the UK Biobank Eye and Vision Consortium

**Affiliations:** 1 Department of Ophthalmology, Kings College London, London, United Kingdom; 2 KCL Department of Twin Research and Genetic Epidemiology, London, United Kingdom; 3 Institute of Ophthalmology, University College London, London, United Kingdom; 4 NIHR Biomedical Research Centre, Moorfield's Eye Hospital NHS Foundation Trust and UCL Institute of Ophthalmology, London, United Kingdom; 5 Department of Public Health and Primary Care, Institute of Public Health, University of Cambridge School of Clinical Medicine, Cambridge, United Kingdom

**Keywords:** genomewide association study (GWAS), chromosome X, intraocular pressure

## Abstract

**Purpose:**

The purpose of this study was to identify genetic variants on chromosome X associated with intraocular pressure (IOP) and determine if they possess any sex-specific effects.

**Methods:**

Association analyses were performed across chromosome X using 102,407 participants from the UK Biobank. Replication and validation analyses were conducted in an additional 6599 participants from the EPIC-Norfolk cohort, and an independent 331,682 participants from the UK Biobank.

**Results:**

We identified three loci associated with IOP at genomewide significance (*P* < 5 × 10^−8^), located within or near the following genes: *MXRA5* (rs2107482, *P* = 7.1 × 10^−11^), *GPM6B* (rs66819623, *P* = 6.9 × 10^−10^), *NDP*, and *EFHC2* (rs12558081, *P* = 4.9 × 10^−11^). Alleles associated with increased IOP were also associated with increased risk for primary open-angle glaucoma in an independent sample. Finally, our results indicate that chromosome X genetics most likely do not illicit sex-specific effects on IOP.

**Conclusions:**

In this study, we report the results of genomewide levels of association of three loci on chromosome X with IOP, and provide a framework to include chromosome X in large-scale genomewide association analyses for complex phenotypes.

Primary open-angle glaucoma (POAG) is the most common form of glaucoma and the leading cause of irreversible blindness worldwide.^1^ Elevated intraocular pressure (IOP) is among the strongest risk factors for POAG; treatment that lowers IOP is effective in slowing POAG progression and vision loss^2^ in both high-tension and normal-tension glaucoma subtypes.^3^ To date, large genomewide association studies (GWAS) have identified over 100 genetic loci associated with IOP,^4^^–^^6^ providing valuable information on POAG genetic etiology. However, as is common for many traits investigated in GWAS,^7^ the role of genetic variation on the X chromosome is unknown.

Observational epidemiologic studies have evidenced sex differences for IOP, with men having a higher mean IOP,^8^^,^^9^ although this difference is not present in all cohorts.^10^^–^^12^ It is possible that the differences between cohorts are a consequence of their statistical power, as the studies that did identify IOP sex differences were larger than those that did not.

Similarly, men have a higher age-adjusted prevalence for POAG (odds ratio = 1.36–1.37),^13^^,^^14^ which would be expected if mean IOP was greater. Additionally, in two longitudinal cohorts, the odds ratio for POAG incidence was approximately 1.3 for men compared with women,^15^^,^^16^ although these were not statistically significant. Despite this, it is worth noting that women account for a greater proportion of the total POAG cases overall, due to having a greater average life expectancy.^17^

The underlying causes for possible sex differences in IOP and POAG are not certain, although there is evidence that hormonal pathways may be a factor. Reduced IOP is associated with estrogen treatment in post-menopausal women^18^^–^^21^ and there is also evidence of association between POAG and testosterone pathways in men.^22^

The unique nature of chromosome X's sex-specific ploidy and differences in the gene expression mechanisms make it an ideal candidate when investigating traits that have, or may have, differences between sexes. However, variants on chromosome X are regularly excluded from GWAS analyses^7^ despite chromosome X being the eighth largest chromosome, containing approximately 5% of human genes.^23^ One factor in the exclusion of chromosome X from GWAS investigations is uncertainty on the need to use chromosome X specific analysis tools. We propose that when studying common polygenic traits, a chromosome X-wide association analysis (XWAS) can be conducted efficiently using the same tools as a conventional autosomal GWAS, and that results from these tests are informative of any X-specific effects on inheritance. We set out to demonstrate that this is possible by conducting an XWAS for IOP to identify, and subsequently replicate, chromosome X variants associated with IOP and POAG risk.

## Methods

### Subjects

All participants provided full informed consent in accordance with ethical approval granted and overseen by the UK Biobank Ethics and Governance Council. Inclusion and exclusion criteria for participants have been described in a previous study,^4^ but briefly all subjects were confirmed to be of West European ancestry through principal component analysis and all first degree relatives, as determined by identity by descent calculations, were excluded. Other exclusion criteria included participants with a history of glaucoma surgery, eye injury, corneal graft surgery, and refractive laser surgery. The top and bottom 0.5 percentiles were also excluded to remove phenotypic outliers. Due to a small increase in errors for chromosome X compared with the autosomes, partly resulting from sex aneuploidies,^24^ genotypic data was available for 102,407 participants, out of the 103,382 participants with post quality control corneal-compensated IOP (IOPcc) data used in the previous autosomal study.^4^

### Phenotyping

Participants were measured once per eye using the Ocular Response Analyzer (ORA; Reichert Corp., Buffalo, NY, USA). Of the two measures of IOP provided by the ORA, we selected the IOPcc measure as it is believed to be a more accurate representation of true IOP, less influenced by corneal properties. The mean of right and left eyes was used as the outcome variable for participants with information for both eyes. If information was only available for a single eye, that measure was used for that individual. POAG disease status was determined using a combination of International Classification of Diseases (ICD-10) codes and self-reported answers to the question “Has a doctor told you that you have any of the following problems with your eyes? (You can select more than one answer)” with “Glaucoma” as one of the possible options. IOP measures were adjusted for participants treated with nonsurgical IOP-lowering medications by dividing the measured IOP by 0.7 (the mean IOP reduction achieved by medication)^25^ to impute the pretreatment value, a method that has been used in previously published IOP GWAS.^4^^,^^26^^,^^27^ A total of 4607 cases were included in the POAG validation analysis, following the exclusion of participants included in the IOPcc analyses. This consisted of 2864 participants that had both an ICD-10 code diagnosis for POAG and self-reported glaucoma. The remaining 1923 cases were self-reported only and had no ICD-10 code for other types of glaucoma. This classification matches previous POAG studies conducted in the UK Biobank.^5^^,^^28^

### Genotyping

Full details for DNA extraction, genotyping, and imputation procedures have been fully described elsewhere.^4^^,^^29^^,^^30^ However, since the previous publication, UK Biobank have addressed the problems with non-Haplotype Reference Consortium (HRC) imputed single-nucleotide polymorphisms (SNPs) and released a corrected dataset. This allowed non-HRC variants to be included in this analysis, providing improved coverage of chromosome X.

### Association Analysis Pipeline

IOPcc was used as the outcome variable in the initial male/female combined analysis in a linear mixed model regression, with an additive model assumption for allelic effects. Male genotypes were coded as homozygous diploid to account for dosage compensation. Adjustments were made for age, sex, and the first five principal components, in addition to a random effects variable to correct for cryptic relatedness and population structure. All IOPcc association analyses in the UK Biobank cohort were performed using BOLT-LMM (version 2.3.2).^31^ The same analysis protocol was used for the sex-stratified analyses (without using sex as a covariate). The POAG validation analysis only included participants that were not included in the IOPcc analyses. POAG disease status was the outcome variable in a mixed model performed using BOLT-LMM, adjustments were made for age, sex, and the first five principal components, in addition to a random effects variable to correct for cryptic relatedness and population structure. POAG disease status was the outcome variable, linear regression betas and respective standard errors were transformed to traditional odds ratios using the formula: log(OR) = β/(µ*(1-µ)), where OR = odds ratio (or respective standard error), β = beta from the linear regression (or respective standard error), and µ = case fraction.

### SNP Heritability

SNP heritability was calculated using a restricted maximum likelihood (REML) analysis with the GCTA software.^32^ The genetic relatedness matrix was calculated using directly genotyped SNPs on chromosome X with GCTA's “make-grm-xchr” option, which is specifically designed to handle the unique chromosome X properties, as with the association analyses, male genotypes were coded as homozygous diploids to account for dosage compensation. Age, sex, and the first five principal components were included as covariates.

### Conditional Analysis

Conditional analysis was performed using the GCTA software.^32^^,^^33^ Only women from the UK Biobank discovery cohort were used as a reference genotype for linkage disequilibrium structure. Conditional analysis is designed for autosomal analysis, however, by using either only male or female samples, this issue is resolved.^34^

### Replication

The Norfolk arm of the European Prospective Investigation into Cancer (EPIC) cohort formed the replication cohort for this study. The recruitment, phenotypic assessment, and genotyping procedures of this cohort have been previously described.^4^^,^^35^^–^^38^ The EPIC-Norfolk cohort was genotyped on the UK Biobank Axiom Array and imputed to the HRC panel.^39^ Principal component analysis was used to ensure all included participants were of European descent. IOPcc was measured three times per eye using the ORA, and the best signal value was selected for each eye prior to calculating the mean of both eyes. This cohort is independent from the UK Biobank. Association analysis in the replication cohort, including 6599 participants and was conducted using a linear regression model performed using the PLINK2 software,^40^ adjustments were made for age, sex, and the first five principal components. Sex-stratified replication was not tested in this cohort due to insufficient statistical power.

## Results

The overall proportion of IOPcc variation (SNP heritability) explained by directly genotyped SNPs (18,058) on chromosome X was 0.7% (standard error [SE] = 0.12%, *P* = 2.7 × 10^−9^). A total of 590,896 genotyped and imputed SNPs were tested for association (the Fig. shows a Manhattan plot of the results), out of which 141 were associated with IOPcc at genomewide significance (*P* < 5 × 10^−8^; Supplementary Table S1), clustered around three discrete genomic regions. The genomic inflation factor for this analysis was λ_GC_ = 1.1, this is consistent with other genetic analyses in large samples. Additionally, we are only reporting results for a single chromosome, and chromosome X is subject to greater linkage disequilibrium than the autosomes as recombination only occurs in women.^41^ Therefore, many of the tested SNPs are not independent of each other, which can lead to an increased λ_GC_ in the absence of genuine inflation (Q-Q plot shown in Supplementary Fig S1). Conditional analysis identified three SNPs (one within each associated region; Table 1) independently associated with IOPcc.

**Figure.**
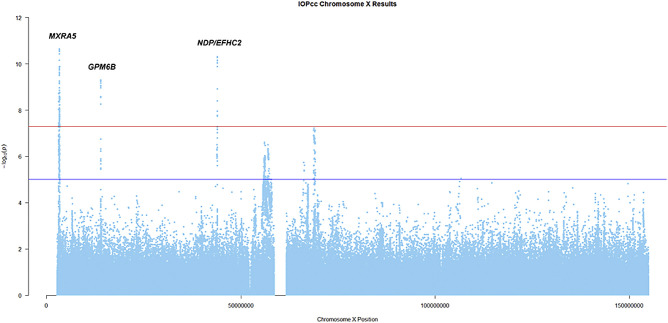
A Manhattan plot of IOPcc association results on chromosome X. The red line is set at the genome-wide significance threshold 5 × 10^−8^. The blue line is set at 1 × 10^−5^ to show suggestive significance.

**Table 1.** Conditional IOPcc SNP Results and Replication

					UK Biobank					EPIC-Norfolk				
SNP	Pos	Locus	A1	Info	Freq	Beta	SE	*P* Value	N	Info	Freq	Beta	SE	*P* Value
rs2107482	3282064	*MXRA5*	G	0.986	0.316	−0.088	0.013	7.1 × 10^−11^	6243	0.962	0.323	−0.15	0.063	0.015
rs66819623	13954397	*GPM6B*	C	0.975	0.572	−0.073	0.013	6.9 × 10^−10^	6092	0.956	0.579	−0.13	0.061	0.026
rs12558081	43939978	*NDP/EFHC2*	A	0.994	0.697	−0.087	0.013	4.9 × 10^−11^	6323	0.966	0.610	−0.15	0.064	0.023

‘SNP’ is the variant rsid, ‘Pos’ is the base pair position (Human genome build 37), ‘Locus’ is the nearest gene/s to the SNP, ‘A1’ is the reference allele, ‘Info’ is a imputation quality score (scale 0-1), ‘Freq’ is the A1 allele frequency in each cohort for this analysis, ‘Beta’ and ‘SE’ are the linear regression coefficients and respective standard errors for the A1 allele in the respective cohort, ‘P’ is the respective association *P* value in each cohort, ‘N’ is the sample size for the respective allele in the EPIC-Norfolk cohort.

The first associated region (based on chromosomal position; rs2107482, *P* = 7.1 × 10^−11^) overlaps with the genomic region coding for the matrix remodeling associated 5 (*MXRA5*) gene. Evidence for association has previously been identified between *MXRA5* and antineutrophil cytoplasmic antibody (ANCA)-associated vasculitis,^42^ which can be associated with severe ocular inflammatory disease and loss of vision.^43^ RNA microarray data shows that *MXRA5* is expressed in both adult and fetal samples of multiple ocular tissues: trabecular meshwork, cornea, and ciliary body.^44^ Notably, the highest relative expression of *MXRA5* was measured in the trabecular meshwork, which accounts for 75% of the resistance to aqueous humour outflow,^45^ a core component in IOP homeostasis. Additionally, *MXRA5* is expressed in fetal optic nerve tissue and continues to be expressed in adult optic nerve tissue at a relatively lower level.^46^

The second associated locus (rs66819623, *P* = 6.9 × 10^−10^) is within an intergenic region, downstream of norrin cystine knot growth factor (*NDP*) and upstream of EF-hand domain containing 2 (*EFHC2*). This region has a low recombination rate in European populations, so the associated SNPs are in linkage disequilibrium with both genes. *NDP* is expressed in several relevant tissues, including the retina and choroid,^47^ the optic nerve,^46^ the trabecular meshwork, and cornea.^44^ Expression of *EFHC2* has also been identified in the trabecular meshwork and cornea.^44^ Expression in ocular tissue, the trabecular meshwork in particular, implicates both genes as plausible functional candidates, although the known relationship of *NDP* with eye development (mutations cause Norrie disease and familial exudative vitreoretinopathy 2 [FEVR2]),^48^ suggest *NDP* could be the more likely functional candidate.

The third associated region (rs12558081, *P* = 4.9 × 10^−11^) was within and around the glycoprotein M6B (*GPM6B*) gene. *GPM6B* is expressed within the trabecular meshwork, cornea and ciliary body^44^; with the highest levels of expression measured in the trabecular meshwork. The functional annotation of *GPM6B* is incomplete, but it likely performs a cellular housekeeping role.^49^ Housekeeping genes tend to have a role in cellular metabolism, and metabolic disorders are a risk factor for elevated IOP and POAG^50^ due to ocular tissues’ relatively high metabolic rate. However, further research into *GPM6B* is required before any conclusions can be drawn on likely mechanisms underlying this association.

Data from the Genotype-Tissue Expression (GTEx) consortium^51^ shows that the conditional SNPs at the *MRXA5* and *GPM6B* loci (rs2107482, arterial tissue, *P* = 1.2 × 10^−13^; and rs66819623, fibroblast cells, *P* = 7.3 × 10^−12^, respectively) are significant expression quantitative trait locus (eQTLs) for their respective genes, although rs66819623 is also an eQTL for the nearby gem nuclear organelle associated protein 8 (*GENIM8*) gene. Neither the conditional SNP nor any other associated SNPs at the *NDP* locus are known eQTLs.

We sought replication in the EPIC-Norfolk cohort, an independent study from the UK Biobank, which has no testing centers in Norfolk, meaning there is little to no sample overlap. As all the SNPs within each associated locus were in strong linkage disequilibrium, we used the three independent SNPs from the conditional analysis, and applied a Bonferroni adjusted replication significance threshold for replication at *P* < 0.017 (0.05/3). Despite the sample size of the replication cohort being less than 6.5% of the discovery cohort, the SNP for the *MXRA5* locus replicated at Bonferroni adjusted significance (rs2107482, *P* = 0.015), whereas the SNPs for the *GPM6B* and *NDP* loci were nominally replicated with the same direction of effect (rs66819623, *P* = 0.026; rs12558081, *P* = 0.023; see Table 1).

Next, we sought secondary validation of the same three independently associated SNPs by testing their association with POAG in the UK Biobank including only participants not included in the original IOPcc analysis, consisting of 4607 cases and 327,075 controls. The SNP at the *GPM6B* locus was significantly associated (rs66819623, *P* = 1.2 × 10^−5^) whereas the SNP at the *NDP* locus was nominally associated (rs12558081, *P* = 0.029), although no significant association was identified for the SNP at the *MXRA5* locus (rs2107482, *P* = 0.16). However, the direction of effect for all three was consistent with their direction of effect on IOPcc (Table 2). These results validate the *GPM6B* locus association, and provide additional support for association of the *MXRA5* and *NDP* loci.

**Table 2.** POAG Validation Results in an Independent UK Biobank Sample

SNP	Pos	Locus	A1	Info	Freq Controls	Freq Cases	OR	Effect	SE	*P* Value
rs2107482	3282064	*MXRA5*	G	0.986	0.317	0.308	0.974	−0.026	0.019	0.16
rs66819623	13954397	*GPM6B*	C	0.975	0.573	0.546	0.926	−0.077	0.018	1.2 × 10^−5^
rs12558081	43939978	*NDP/EFHC2*	A	0.994	0.697	0.684	0.960	−0.041	0.019	0.029

‘SNP’ is the variant rsid, ‘Pos’ is the base pair position (Human genome build 37), ‘Locus’ is the nearest gene/s to the SNP, ‘A1’ is the reference allele, ‘Info’ is a imputation quality score (scale 0-1), ‘Freq controls’ is the A1 allele frequency in the controls and ‘Freq cases’ is the A1 allele frequency in POAG cases in the UK Biobank cohort for this analysis, ‘OR’ is the odds ratio for A1, ‘Effect’ and ‘SE’ are the natural logarithm of OR and the respective standard error, ‘P’ is the respective association *P* value.

We next conducted a sex-stratified XWAS for IOPcc in the UK Biobank discovery cohort. In the men-only analysis, the amplitude and direction of the associations remained practically unchanged, compared to the mixed-sex analyses reported before, although the statistical significance tended to decrease, presumably due to the halved sample size of the men-only participants (Table 3). In the women-only analysis, while effect sizes were reduced (but comparable to the male-only analysis), association significance tended to be markedly reduced compared to the male analysis, despite a similar sample size for both. Despite the stark differences in significance of associations, the effect estimates for two loci (*MXRA5* and *NDP*) were similar between men and women and fell well within each other's 95% confidence interval. Meanwhile, the effect estimate for women at the *GPM6B* locus was half of the effect estimate for men (see Table 3).

**Table 3.** IOPcc Sex-Stratified Results

			Men						All Women						Homozygous Women					
SNP	Locus	A1	*N*	Freq	Beta	SE	95% CI	*P* Value	N	Freq	Beta	SE	95% CI	*P* Value	N	Freq	Beta	SE	95% CI	*P* Value
rs2107482	*MXRA5*	G	48015	0.317	−0.098	0.017	−0.065 −0.129	1.9 × 10^−8^	54392	0.315	−0.073	0.021	−0.032 −0.114	7.5 × 10^−4^	31328	0.180	−0.093	0.025	−0.044 −0.142	1.7 × 10^−4^
rs66819623	*GPM6B*	C	48015	0.572	−0.086	0.016	−0.055 −0.117	4.2 × 10^−8^	54392	0.571	−0.045	0.020	−0.006 −0.084	0.017	28775	0.635	−0.046	0.021	−0.005 −0.087	0.028
rs12558081	*NDP/EFHC2*	A	48015	0.696	−0.092	0.017	−0.059 −0.125	1.4 × 10^−7^	54392	0.698	−0.084	0.022	−0.041 −0.127	1.5 × 10^−4^	31324	0.844	−0.068	0.026	−0.017 −0.119	8.8 × 10^−3^

‘SNP’ is the variant rsid, ‘Locus’ is the nearest gene/s to the SNP, ‘A1’ is the reference allele, ‘Freq’ is the A1 allele frequency for each subgroup in this analysis, ‘Beta’ and ‘SE’ are the linear regression coefficients and respective standard errors for the A1 allele in the respective sample, ‘95% CI’ is the 95% confidence interval for the Beta, ‘P’ is the respective association *P* value in each sample, ‘N’ is the respective sample size of each sample. The homozygous female sample is defined by female participants that are homozygous for the tested SNP on each row.

To test whether the difference of significance between the analyses for men and women is a consequence of skewed X-inactivation causing increased phenotypic variance in heterozygous women, we tested the association of the same SNPs in women that were homozygous for each conditional SNP (see Table 3). Despite a loss of almost half the sample size when compared to the all-women analysis, the conditional SNP for the *MXRA5* locus was more significant in the homozygous women analysis (rs2107482, *P* = 1.7 × 10^−4^), whereas significance for the other two loci was similar between the two analyses despite the large difference in sample size (see Table 3).

Interestingly, the effect size for SNPs at the *GPM6B* locus in both the all-women and the homozygous women analyses was half that of the all-men analysis. Previous research using GTEx data showed *GPM6B* escapes X-inactivation^52^; the difference in effect size between men and women in our analyses is most likely a consequence of this, as a haploid male with one expressed copy of the effect allele will have the same phenotypic outcome as a female homozygous with two expressed copies.

## Discussion

This is the first XWAS conducted in the study of IOP, as previous studies have concentrated on autosomes. It is also the first study to calculate the IOPcc SNP heritability for chromosome X (0.007), which accounts for approximately 2.8% of the total SNP heritability, increasing the total proportion of IOPcc variation explained by common SNPs to 0.254.

Three loci were found for IOP on the X chromosome, and the strongest locus was between *NDP* and *EFHC2.* Based on all currently available information, there is evidence for *NDP* as a candidate gene for association at that locus. A key component of this evidence is *NDP*'s role in eye development and the Mendelian diseases Norrie disease and FEVR2^48^; clinical features of these diseases include abnormal retinal vascular development and angiogenesis. Changes in ocular angiogenesis could potentially influence IOP as aqueous humour drains through the uveoscleral route via blood vessels.^53^ Clinical signs of these diseases can also include iris synechiae and a shallow anterior chamber,^54^^,^^55^ more typically viewed as risk factors for primary angle closure glaucoma (PACG)^56^^,^^57^ rather than POAG or ocular hypertension. Although PACG and POAG are clinically distinct, recent well-powered genetic studies have identified shared genetic risk between IOP and both PACG and POAG,^4^ thus association at the *NDP* locus in our study further supports evidence that some mechanisms underlying PACG also contribute to normal variation in IOP.

Replication had limitations due to sample size, IOPcc was tested in a sample size less than 6.5% of the discovery cohort and the POAG validation dataset had 4607 POAG cases providing an effective sample size of 9086, less than 10% of the discovery sample. Despite this, by using a combination of direct IOPcc replication and POAG validation tests in two independent samples, we provide evidence that the three loci identified in this study are true positive associations.

Results from our sex-stratified analyses indicate that for the *NDP* and *MXRA5* loci there are not sex-specific effects. The similarity in betas with differences in *P* values for the *MXRA5* and *NDP* loci suggests these loci have the same effect in men and women, but that there is more uncertainty or statistical “noise” in the female analysis. Men have only one copy of the X chromosome, resulting in the same allelic effects being present across all cells within a tissue; women have two copies, although for the majority of X genes the copy on one of the chromosomes is silenced by X-inactivation.^52^ This causes each cell to only express the allelic effects from one of the X chromosomes with all the cells within a tissue being a mosaic of cells expressing each. The choice of which X is inactivated is generally random resulting in an average 50:50 pattern across cells within a tissue^58^; however, this is not always the case and the ratio of expression can be highly skewed with the majority of cells within a tissue expressing the same copy of X, known as “skewed X-inactivation.” The degree of skewed X-inactivation is variable both between and within individuals, and varies with age, smoking status, cell type, sub-cell type, and disease status.^59^ The large variation in skewed X-inactivation means that whereas the linear model in our XWAS codes female heterozygotes as having one copy of the effect allele, there will be much variability in the relative expression and effective gene dosage, depending on the degree of skewed X-inactivation in the IOP relevant tissues. This increased variance in heterozygotes will cause an increase in the standard error for the linear regression during association analysis, and thus a less significant *P* value, whereas the slope/estimate of effect size will be relatively unchanged as the homozygotes at each end of the slope will be the same. This indicates that there are not sex-differences for the association between the *MXRA5* and *NDP* loci with IOPcc, but that greater statistical power is available in men, due to skewed X-inactivation in heterozygous women. Therefore, when studying chromosome X for complex traits, it is to be expected that the male participants will drive the associations in an XWAS and be the primary source for identifying associated loci, but female samples are still required to determine whether there are any sex-specific effects in allele effect size.

There is a difference in allelic effect size for associated variants at the *GPM6B* locus, with the allelic effect size for homozygous women being approximately half that of the effect size in men. *GPM6B* escapes X-inactivation in women,^52^ and women are effectively diploid at this locus whereas men are haploid, therefore, homozygous women will effectively have the same overall phenotypic effect as men carrying the risk allele.

Although there is evidence for sex-specific effects for IOP variation and POAG risk,^8^^,^^9^^,^^13^^–^^16^ our results indicate that chromosome X genetics are most likely not a factor for any sex-specific effects. Previous literature indicates hormonal pathways as the most probable candidate for these effects^18^^,^^19^^,^^22^^,^^60^ and androgen receptors are expressed in multiple ocular tissues.^61^

The initial combined XWAS begins with the assumption that men have full dosage compensation for chromosome X genes, allowing the use of “off the shelf” analysis software such as BOLT-LMM^31^ just as with autosomal analysis. The subsequent sex-stratified and homozygous female tests confirm whether this assumption is valid or not for each associated locus. Utilizing this pipeline allows for quick and efficient association analysis of chromosome X, whereas accounting for the unique properties that separate it from the autosomes. A software toolset has previously been developed specifically for conducting an XWAS,^62^ which includes tests for increased variance in heterozygous women, but, unfortunately, a mixed model is not currently implemented in the association testing. There is considerable cryptic relatedness within the UK Biobank, with 30.3% of participants related (third-degree or closer) with at least one other participant.^29^ Therefore, while very closely related participants were removed during our quality control (QC) process, a linear mixed model is still required for analysis in this cohort to correct for the family structure and prevent inflation of results.

In summary, our analysis identified three novel loci associated with IOPcc and POAG risk. One of these loci might provide supportive evidence of some shared genetic architecture between PACG and normal IOP variation. This analysis indicates that despite sex differences in chromosome X ploidy, this does not result in sex differences in the genetic risk from chromosome X variants for this trait. Finally, we demonstrate that chromosome X can be included in GWAS conducted in very large datasets, with minimal effects to the analysis time and computational burden.

## Supplementary Material

Supplement 1

Supplement 2

Supplement 3
