# Supplementary figures and images for: The Role of Chromosome X in Intraocular Pressure Variation and Sex-Specific Effects
Source: Invest Ophthalmol Vis Sci. 2020 Sep 14;61(11):20. doi: 10.1167/iovs.61.11.20 (PMC7490223; doi:10.1167/iovs.61.11.20)

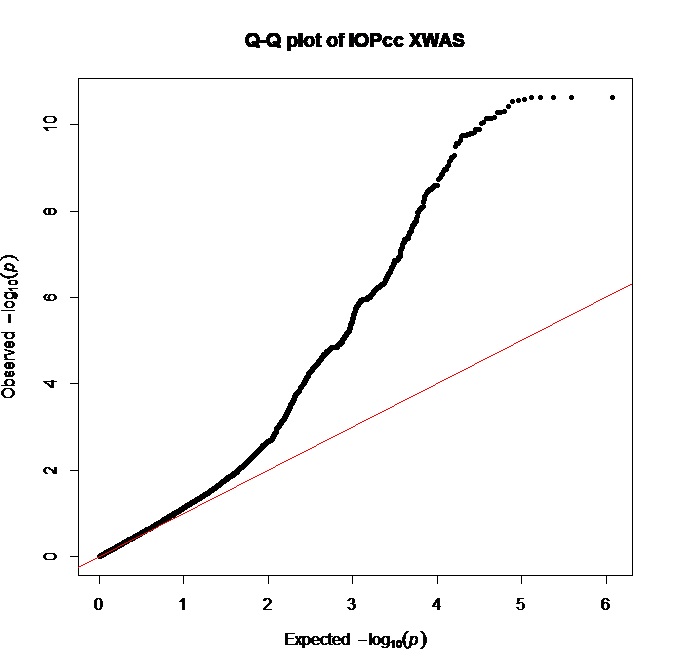

Supplement: Supplement 2 [file iovs-61-11-20_s002.jpg]
